# Majeed Syndrome: A Review of the Clinical, Genetic and Immunologic Features

**DOI:** 10.3390/biom11030367

**Published:** 2021-02-28

**Authors:** Polly J. Ferguson, Hatem El-Shanti

**Affiliations:** Stead Family Department of Pediatrics, University of Iowa Carver College of Medicine, 200 Hawkins Drive, Iowa City, IA 52242, USA; hatem-el-shanti@uiowa.edu

**Keywords:** majeed syndrome, LPIN2, LIPIN2, chronic non-bacterial osteomyelitis, chronic recurrent multifocal osteomyelitis, autoinflammatory, inflammasome, macrophage, osteoclast

## Abstract

Majeed syndrome is a multi-system inflammatory disorder affecting humans that presents with chronic multifocal osteomyelitis, congenital dyserythropoietic anemia, with or without a neutrophilic dermatosis. The disease is an autosomal recessive disorder caused by mutations in *LPIN2*, the gene encoding the phosphatidic acid phosphatase LIPIN2. It is exceedingly rare. There are only 24 individuals from 10 families with genetically confirmed Majeed syndrome reported in the literature. The early descriptions of Majeed syndrome reported severely affected children with recurrent fevers, severe multifocal osteomyelitis, failure to thrive, and marked elevations of blood inflammatory markers. As more affected families have been identified, it has become clear that there is significant phenotypic variability. Data supports that disruption of the phosphatidic acid phosphatase activity in LIPIN2 results in immune dysregulation due to aberrant activation of the NLRP3 inflammasome and overproduction of proinflammatory cytokines including IL-1β, however, these findings did not explain the bone phenotype. Recent studies demonstrate that *LPIN2* deficiency drives pro-inflammatory M2-macrophages and enhances osteoclastogenesis which suggest a critical role of lipin-2 in controlling homeostasis at the growth plate in an inflammasome-independent manner. While there are no approved medications for Majeed syndrome, pharmacologic blockade of the interleukin-1 pathway has been associated with rapid clinical improvement.

## 1. Introduction

### 1.1. Autoinflammatory Disorders

Autoinflammatory diseases present with recurrent or persistent inflammation that occurs in the absence of self-reactive T or B cells which distinguishes them from autoimmune disorders [1,2]. Instead, it is the innate immune system that is dysregulated and that leads to persistent or recurrent bouts of inflammation. The major breakthroughs in the understanding of autoinflammatory diseases came around the turn of the century with the identification of the genetic cause for several periodic fever syndromes, including familial Mediterranean fever (FMF) due to pathogenic variants in *MEFV* and cryopyrin associated periodic syndrome (CAPS) due to pathogenic variants in *NLRP3*, affecting the pyrin and NLRP3 inflammasome function, respectively, and leading to dysregulation of IL-1 production [3,4,5] Since that time, there has been a flurry of discovery in the autoinflammatory disorder field [6]. These disorders can affect many organ systems including a subgroup of autoinflammatory disorders that target the bone [7,8,9]. Their unifying features are sterile osteomyelitis or osteitis of one or more bones, often accompanied by inflammatory disorders of the skin and gastrointestinal tract. 

### 1.2. Chronic Recurrent Multifocal Osteomyelitis (CRMO)

The most common autoinflammatory bone disorder is chronic recurrent multifocal osteomyelitis (CRMO) which predominantly affects children with an average age of onset around 9 to 10 years and presents with bone pain with or without associated swelling [10,11,12,13,14]. It was first described by Giedion et al. in 1972 as a symmetric, sterile, multifocal osteomyelitis [15]. An alternative name is chronic non-bacterial osteomyelitis (CNO) which has been proposed as an umbrella term as some cases remain unifocal and not all patients have recurrent disease [8,16,17]. When adults present with a similar disease phenotype, the term synovitis, acne, pustulosis, hyperostosis and osteitis (SAPHO) syndrome is used [8,18,19,20,21]. Individuals with CRMO and their close relatives are more likely to have or develop inflammatory bowel disease (most often Crohn disease), psoriasis or inflammatory arthritis suggesting a shared pathogenetic process [10,22,23,24,25,26,27]. Most cases of CRMO are sporadic but there are cases of affected siblings, concordant monozygotic twins and parent-child duos supporting a genetic contribution to the etiology of the disease [10,28,29,30]. A putative susceptibility locus was reported on chromosome 18q21.3–18q22 but this was not reproduced in a larger cohort when sequencing rather than microsatellite marker analysis was used to assess for linkage [30,31]. CRMO can also occur as part of a Mendelian syndrome in which a single gene defect leads to sterile multifocal osteomyelitis further supporting a genetic contribution to the etiology of the disease [32,33,34,35,36,37]. 

The molecular mechanisms of non-syndromic CRMO remains unclear but the existing data suggests that it is a genetically complex disorder leading to an imbalance between pro- and anti-inflammatory cytokines produced by innate immune cells, ultimately leading to osteoclast activation with osteolytic destruction of the bone [9]. Despite the phenotypic similarities to bacterial osteomyelitis, culture of the bone is typically negative, antibiotics do not produce sustained improvement and bacterial DNA is not detected by molecular methods [10,16]. Proinflammatory cytokines including IL-1β, IL-6 and TNF are elevated in the blood of CRMO patients whereas ‘anti-inflammatory’ or immunoregulatory cytokines including IL-10 are reduced [9,38,39,40,41,42]. Peripheral blood monocytes from patients with CRMO produce lower levels of IL-10 and IL-19 when stimulated with lipopolysaccharide versus control monocytes [42]. Evaluation of human CRMO bone specimens for cytokines suggests dysregulation of IL-1 pathway but this data is from a few small studies [9,43]. The proinflammatory cytokines found in the blood of these patients are known to activate osteoclasts via receptor activator of nuclear factor kappa-Β (RANK) and RANK ligand signaling which could lead to bone resorption producing the osteolytic lesions. Epigenetic alterations have also been found in CRMO including reduced phosphorylation of histone H3 at position 10 (H3S10P) which would result in ‘closure’ of the IL-10 promotor leading to reduced IL-10 transcription [44]. 

There are multiple animal models of the disease including humans, mice, rats, dogs and non-human primates [45,46,47,48,49,50,51] several of which have been shown to be genetically driven [31,33,36,38,49,52,53,54,55,56,57,58,59]. Utilizing murine models of CRMO, including the proline-serine-threonine phosphatase interacting protein 2 (PSTPIP2) deficient and Ali18 models, it has been shown that the disease is a hematopoietically-driven innate immune system disorder, occurring independent of the adaptive immune system [37,38,55,56]. Furthermore, work on the PSTPIP2 deficient cmo mouse has shown that it is an IL-1β driven disorder with granulocytes, particularly neutrophils playing a key role in the inflammatory process [49,60,61,62,63]. Due to these features, CRMO has been classified as an autoinflammatory disorder [2,22]. 

### 1.3. Autoinflammatory Bone Disease Syndromes

There are two syndromic forms of CRMO in humans that have also been shown to be IL-1 mediated disorders by in vitro investigations and by favorable responses to IL-1 blocking drugs [34,35,64,65,66]. One is Majeed syndrome which is the focus of this review. The other is the deficiency of the interleukin-1 receptor antagonist (DIRA) in which, sterile multifocal osteomyelitis/osteitis and skin pustulosis are the dominant phenotypes [34,35,67,68,69,70,71,72,73,74,75,76,77,78]. Patients with DIRA present in infancy with systemic inflammation, severe multifocal osteomyelitis and pustulosis of the skin. It is caused by either deficiency or loss of function mutations in the gene that encodes the IL-1 receptor antagonist leading to unfettered IL-1 signaling and results in a systemic inflammatory disorder that if left untreated is often fatal [34,35,67,68,69,70,71,72,73,74,75,76,77,78]. While a direct connection to IL-1 signaling is evident in DIRA, that connection had been less clear in the Majeed syndrome which is caused by pathogenic variants in *LPIN2*, in which the encoded protein plays a central role in lipid metabolism. However, clinical observations and basic research has confirmed that Majeed syndrome is also an IL-1 mediated disease. We review the clinical, genetic and immunologic features of Majeed syndrome and the recent data that links the derangements in lipid metabolism with the innate immune system dysfunction. 

## 2. Majeed Syndrome

Majeed syndrome was first described in 1989, in three children from a consanguineous two-related-sibship family who presented with CRMO and a congenital dyserythropoietic anemia (CDA) that was characteristically microcytic [33]. This was followed with the addition of a fourth child [54] and the description of two siblings from another unrelated consanguineous family [54]. Two brothers from the initial family also had a neutrophilic dermatosis, Sweet syndrome in addition to CRMO and CDA [33]. The children were severely affected and had significant failure to thrive and growth delay [53]. Each child presented in the first 2 years of life with recurrent fevers and bone pain, sometimes with subsequent joint contractures due to severe multifocal sterile osteomyelitis, and often with hepatosplenomegaly [53]. All affected individuals had CDA that required multiple red blood cell transfusions and partially resolved in one patient after a splenectomy [33,53,54]. 

Inflammatory markers, including the erythrocyte sedimentation rate, were elevated in all 6 children [53]. Non-steroidal anti-inflammatory medications improved but did not control symptoms. Since the disease segregated in an autosomal recessive pattern, *LPIN2* was identified as the responsible gene using homozygosity mapping and positional cloning [52]. To date, only 24 individuals from 10 families with molecularly confirmed Majeed syndrome have been reported in the literature (Table 1) [33,52,53,54,64,79,80,81,82,83,84,85]. 

### Clinical Features of Majeed Syndrome

Based on the earliest reports, Majeed syndrome became recognizable by the clinical triad of early onset CRMO, severe CDA and a neutrophilic dermatosis [53]. However, as more cases have been reported it has become evident that < 10% of those affected have all 3 features (Table 1 and Table 2). Most individuals with Majeed syndrome present in the first 2 years of life and 91% have both CRMO and CDA, and the neutrophilic dermatosis, if present, may be transient. The median age at onset is 12 months old (average age of onset = 20.4 months), with CRMO being the prominent phenotype in Majeed syndrome with 91 percent having radiologic or clinical evidence of CRMO (only 2 individuals had no musculoskeletal symptoms but were not imaged). 

The CRMO of Majeed syndrome presents with recurrent episodes of bone pain affecting the long bones, often close to joints, with a predilection for the lower extremities. Unlike classic CRMO, involvement of the mandible, clavicle, anterior chest, spine and pelvis have not been reported, however, whole body magnetic resonance imaging has only been performed in a few Majeed syndrome patients. Seven individuals have had bone biopsies with six demonstrating culture negative osteomyelitis and the seventh demonstrating osteonecrosis [33,64,79,83,86]. 

The diagnosis of Majeed syndrome requires a high index of suspicion as about half of patients had objective changes on physical examination such as swelling or warmth overlying the involved bone or nearby joint. 

The anemia of Majeed syndrome is hypochromic and microcytic and ranges in severity from very mild to quite severe with about a quarter of patients requiring red blood cell transfusions [33,80,84,86]. Bone marrow aspiration or biopsy demonstrates morphological abnormalities, such as erythroid hyperplasia with binuclearity or multinuclearity, which is typical of CDA [53,54,80,84,86]. Based on bone marrow morphology, associated anomalies and molecular etiology, the CDAs have been recently classified into five categories: CDAI, CDAII, CDAIII, transcription-factor-related CDA and CDA variants, with the CDA of Majeed syndrome falling in the last category [87]. While CRMO and CDA are commonly reported in Majeed syndrome, only 2 of 24 affected individuals had skin disease, both with the neutrophilic dermatosis Sweet syndrome [53]. There are two other reports of a patient with CRMO and Sweet syndrome in the absence of CDA, as a bone marrow biopsy was normal in one [87,88]. Thus, it remains to be determined if Sweet syndrome is part of Majeed syndrome. 

Elevated blood inflammatory markers were present in 21 of 21 patients tested; this likely contributes to the failure to thrive, growth delay and organomegaly that are present in ~ 30% of affected individuals. Nearly half of the patients have recurrent fevers and for many this is one of the first manifestations of the disease, therefore, Majeed syndrome should be in the differential diagnosis of the periodic fever syndromes. While the initial reports of Majeed syndrome were of early onset severe CRMO and severe CDA, it has become evident that there is considerable phenotypic variability in the presentation. Moussa et al., Pinto-Fernandez et al. and Rao et al., reported children where the age of onset of disease was 4, 6 and 8 years, respectively [83,84,86]. Even more pronounced phenotypic variation was described by Roy et al. in a family of 6 who were all homozygous for a novel mutation in *LPIN2* for which 3 had a typical severe phenotype while 2 had mild symptoms and one adult had only mild knee pain throughout his childhood and adult life [85]. 

## 3. Genetics of Majeed Syndrome

**Figure 1:**
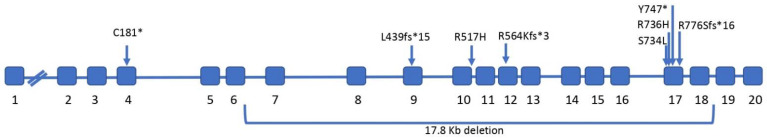
Majeed syndrome associated mutations. *LPIN2* is composed of 20 exons. Pathogenic mutations are located throughout the gene. Most disease-causing mutations reported to date are predicted to results in early truncation or in exon deletion. Two missense mutation. The in vitro functional work by Donkor et al. showed that the mutant protein is expressed but lacked PAP activity.

The observations that males and females are equally affected with unaffected consanguineous parents and the presence of affected individuals from related sibships highly suggest that Majeed syndrome is a distinct entity with an autosomal recessive mode of inheritance [53]. Homozygosity mapping utilizing DNA from 2 unrelated affected families demonstrated linkage with markers in a 1.8 Mb region on chromosome 18p. Sanger sequencing of genes in the candidate interval revealed unique mutations in *LPIN2* in each family [52]. Affected individuals from one family were homozygous for a missense mutation replacing the highly conserved serine at amino acid 734 with a leucine (p.S734L), while affected children from the other family had a frameshift mutation caused by a consecutive 2 base pair deletion resulting in a premature stop codon in the first quarter of the coding sequence (p.C181*) [52]. To date, 9 unique mutations have been reported in families of Arabic, Turkish, Chinese, Indian (East Asia), Pakistan, Spanish and mixed-race backgrounds (Figure 1). These come from 10 unrelated families (the reports by Al-Mosawi et al. in 2007 and 2019 are from the same extended family). All patients reported to date have been homozygous with only two patients having a compound heterozygous genotype [81,82]. Most of the mutations are deleterious mutations predicted to lead to a truncated protein and loss of function. However, two families harbor missense mutations changing the highly conserved amino acids; one altering the serine at amino acid 734 to a leucine (p.S734L) and the other altering proline at amino acid 736 to a histidine (p.P736H) [33,52,85]. To date, there is no clear genotype-phenotype correlation pattern providing an explanation to the clinical heterogeneity of Majeed syndrome. 

It is of note that a recent study found five out of 182 Turkish FMF patients are heterozygous for four variants in LPIN2, three variants are previously described and are likely benign, and the fourth is a novel nonsense variant, p.Y732X [89]. Further, another report details a child diagnosed clinically with FMF and CDA who underwent successful bone marrow transplant for severe anemia. In addition to the anemia and fevers, that child also had bone pain and limb swelling. The child was heterozygous for pathogenic mutation p.M680I but a second disease associated mutation in *MEFV* was not found. This case report predates the discovery of LPIN2 as the disease causing mutation in Majeed syndrome and while the authors speculate that FMF and CDA II were co-segregating in this patient, we posit that the child likely had Majeed syndrome given the limb pain, limb swelling, marked inflammatory markers and CDA [90]. 

### 3.1. Pathogenesis of Majeed Syndrome

The LIPIN family (LIPIN1, LIPIN2, and LIPIN3) is a trio of cytosolic intracellular proteins with phosphatidic acid phosphohydrolase (PAP) activity that converts phosphatidic acid to diacylglycerol (DAG) [89,90,91]. This is the penultimate step in triacylglycerol (TAG) synthesis and a regulatory nodal point that can impact synthesis of phosphatidylcholine, phosphatidylethanolamine, and other membrane lipids. Beyond this central role in lipid metabolism, the LIPINs can regulate lipid intermediates in cellular signaling pathways and have transcriptional co-regulatory capabilities [90,92,93]. These proteins have been shown to be involved in a wide range of cellular processes including autophagy, inflammation and as a regulator of gene expression [52,65,66,90,92,94,95,96,97]. Mutations in LPIN1 and LPIN2 cause disease in mouse and humans with desperate phenotypes which is likely influenced by their differences in tissue expression and as well as by compensatory mechanisms. While tissue expression is broad in all of the LIPINs, LIPIN1 is most highly expressed in skeletal muscle, adipose tissue, peripheral nerve and testis, whereas LIPIN2 is the most abundant LIPIN in the liver, small intestine, macrophages and CNS [98]. The distribution of LIPIN3 overlaps with that of the other LIPINs but isn’t the dominant protein in any tissue [98]. 

Deficiency of LPIN1 has significant consequences in adipose tissue, liver, skeletal muscle and nervous system [90,99,100]. *Lpin1* mutations where identified in a spontaneous mutant mouse called the fatty liver dystrophy or fld mouse [99,101,102]. The fld mouse has a phenotype consisting of lipodystrophy, a transient neonatal fatty liver and progressive non-inflammatory peripheral demyelinating neuropathy [102,103]. LPIN1 is highly expressed in skeletal muscle, yet these mice do not have an overt muscle phenotype under normal colony conditions but have abnormalities in skeletal muscle when stressed and have subtle skeletal muscle fiber changes seen on histopathologic exam [98,104]. This is in contrast to the phenotype seen in humans with homozygous or compound heterozygous loss of function mutations in LPIN1 which cause severe episodes of recurrent myoglobinuria [100]. Affected individuals have severe episodes of muscle necrosis (rhabdomyolysis) that causes profound muscle weakness accompanied by myoglobinuria which has led to renal failure and is potentially fatal [105,106,107,108]. Despite the severe phenotype during attacks, affected individuals are well in between episodes. The precise triggers have not been identified but the episodes are most often associated with febrile illnesses which implicates infections as a possible precipitating factor. Surprisingly, the episodes of myoglobinuria are rarely related to exercise. Unlike the murine models of Lpin1 deficiency, affected humans do not have lipodystrophy as part of the phenotype. The lack of lipodystrophy has been postulated to be from redundancy in the roles the LIPIN proteins have in triacylglycerol synthesis in human adipocytes [109]. Genetic dissection demonstrated that it is the lack of Lipin1 PAP activity, rather than co-activator activity, that drives the altered lipid metabolism that results in lipodystrophy in mice [110,111,112]. Thus, PAP activity is needed for normal adipocyte differentiation and triacylglcerol synthesis.

There are also key differences in the phenotypes seen in Majeed syndrome and that seen in *Lpin2* knockout mice. Mice deficient in Lipin2 do not develop sterile osteomyelitis by gross inspection and have normal bone histopathology and no osseous lesions by radiography; thus, they are missing a key feature of Majeed syndrome [113]. Yet there is phenotypic similarity. Similar to humans, *Lpin2* knockout mice develop anemia with features consistent with a congenital dyserythropoietic anemia [113]. The anemia in mice is mild, whereas, humans with Majeed syndrome the anemia is of variable severity from mild and asymptomatic to severe anemia that requires recurrent red blood cell transfusions [54]. Another difference is that there are neurologic abnormalities in Lipin2 deficient mice which have not been reported in Majeed syndrome including the development of tremor, ataxia and difficulties with maintaining their balance in the mice beginning around age 5 to 6 months [113]. Both Lipin1 and Lipin2 proteins are present in the cerebellum in young mice but with age, Lipin1 expression falls to undetectable levels in wildtype and Lipin2 knockout mice. This suggests that Lipin1 is able to compensate for the lack of Lipin2 in the cerebellum in young mice but that as the mice age, the lack of Lipin 1 and 2 leads to cerebellar dysfunction [113]. There is additional evidence for redundancy in the system as Lipin2 deficient mice do not have abnormal lipid homeostasis on a chow diet which is associated by a compensatory increase in hepatic Lipin1. However, stressing the mice by feeding them a high fat diet does result in lipid dysregulation. No abnormalities in fat metabolism have been identified in Majeed syndrome but investigations have been limited to analysis of blood lipids in a few children [80]. It is likely that a compensatory mechanism for lipid homeostasis is also occurring in humans with Majeed syndrome but this has not been experimentally proven. There is no information about long term effects of LIPIN2 deficiency on the central nervous system function in humans as the original cohorts described by Majeed et al. have been lost to follow up and the subjects in other reported cases are still too young to determine if cerebellar dysfunction will occur with age [33,53,54]. The importance of compensatory mechanisms in the Lipin family of proteins is demonstrated by embryonic lethality in mice that are deficient in both *Lpin1* and *Lpin2* [113]. To date, mutations in *LPIN3* have not been linked to human or murine disease.

Five of the 9 *LPIN2* mutations identified in affected individuals with Majeed syndrome are predicted to cause an early termination codon and a truncated protein, if the mRNA escapes the nonsense-mediated RNA decay and one is a whole gene deletion. It is unclear if any LIPIN2 protein is produced in patients with these pathogenic variants. Three of the 9 pathogenic variants are single nucleotide substitutions resulting in non-synonymous variants (p.S734L, p.P736H and p.R517H) [52,85]. The first two amino acid substitutions are separated by one amino acid, which implies that the disruption of protein function in this region of the LIPIN2 is important in the pathogenesis of Majeed syndrome. Donkor et al. analyzed the functional consequences of the human p.S734L in a mouse model [114]. The in vitro functional work showed that the mutant protein is expressed but lacked PAP activity [114]. They also demonstrated that the mutation did not disrupt Lipin association with microsomal membranes. Further they demonstrated that, similar to Lipin1, Lipin2 can act as a transcriptional coactivator for peroxisome proliferator-activated receptor-response elements and that the co-activator function is not disrupted by the p.S731L (equivalent to human p.S734L) mutation in their murine model [114]. This study strongly implicates LIPIN2 PAP activity in the pathogenesis of Majeed syndrome. Valdearcos et al. demonstrated that LIPIN2 is important in controlling inflammation triggered by excess saturated fatty acids in vitro [66]. They under-expressed *LPIN2* in murine and human monocytes and found that the monocytes produce excess proinflammatory cytokines, including TNF and IL-6, when exposed to excessive amounts of the saturated fatty acid palmitic acid. In contrast, they found that when *LPIN2* is over-expressed in the same experimental setting that the inflammatory response to palmitic acid was blunted [66]. However, the connection between diminished PAP activity and inflammatory bone disease remained unclear.

We and others have shown that the pro-inflammatory cytokine IL-1 drives the inflammatory bone disease and systemic inflammation in Majeed syndrome [64]. Therapeutically blocking the IL-1RI or IL-1β (*n* = 10), but not TNF (*n* = 4), results in prompt resolution of systemic inflammation and healing of the sterile osteomyelitis seen in Majeed syndrome patients (Table 2) [64,79,81,83,85,86]. There is additional evidence that dysregulated IL-1 mediated signaling is central to the pathogenesis of sterile osteomyelitis. Pstpip2 deficient cmo mice develop kinked tails, paw deformities and inflammation of the skin and soft tissues of the ears [10,11]. Immune dysregulation is present with the over production of inflammatory cytokines including IL-6 and macrophage inflammatory protein 1- alpha (MIP-1α) accompanied by increase production of IL-1 in the bones accompanied by increased osteoclast function [12,13]. Bone marrow transfers in this model demonstrate that the disease is hematopoietically driven [14]. Genetic crosses demonstrate that it is innate immune system driven as the adaptive immune system is dispensable [14]. The disease is completely blocked in cmo mice that lack an IL-1 receptor [13,15] and the effect of IL-1 is mediated through IL-1β rather than IL-1α [13,15]. While our group demonstrated that the disease can occur independent of the Nlrp3 inflammasome, others propose that the Nlrp3 inflammasome via caspase-1 and caspase-8 redundancy may play a role in disease. The Src family kinases are also involved in aberrant signaling that can cause sterile osteomyelitis in both the cmo mouse which is dependent on function SYK [16] and in the Ali18 mouse which is due to gain of function mutations in FGR [17]. 

### 3.2. Majeed Syndrome as an Inflammasomopathy

There are several IL-1 mediated human autoinflammatory disorders that are caused by genetic mutations in inflammasome components [115,116,117,118]. Given that Majeed syndrome is also IL-1 driven, investigators set out to determine if Majeed syndrome is an inflammasomopathy. Inflammasomes are intracellular macromolecular protein complexes that assembles in response to numerous stimuli (including various pathogens, uric acid crystals, certain lipids, and signal cellular stress molecules such as ATP) ultimately leading to IL-1 production and release. The NLRP3 inflammasome in macrophages requires 2 signals for activation and assembly including a priming step such as LPS binding to Toll-like receptors and a second signal such as ATP binding to the purinergic P2X7 receptor leading to efflux of intracellular K+ and subsequent inflammasome assembly. Given the role of IL-1 in Majeed syndrome and its phenotypic characteristics as an autoinflammatory disorder. Lorden et al. performed a series of experiments that established the important role of the NLRP3 inflammasome in the excess IL-1β production that occurs in the absence of LIPIN2. Using both human and murine in vitro systems, they demonstrated that there is enhanced production of IL-1β by Lipin2 deficient macrophages [65]. Further, that Lipin2 regulates MAPK activation, inhibits activation and sensitization of the purinergic P2X7 receptor, inhibits inflammasome assembly, and controls caspase-1 activation [65]. They showed that intracellular cholesterol levels were low in bone marrow derived macrophages from Lipin2 deficient mice and when Lipin2 is silenced in RAW264.7 cells, and further, that low intracellular cholesterol levels affected P2X7R function. Normalization of intracellular cholesterol in Lipin2 deficient cells normalized P2X7R function and reversed inflammasome overactivation. Lastly, they confirmed that LIPIN2 restrains inflammasome assembly and activation in vivo using *Lpin2* deficient mice [65]. Collectively, this provides support for Majeed syndrome being an NLRP3 inflammasomopathy. Further studies are needed to fully understand the interplay of cellular lipid alterations and immune function.

**Figure 2:**
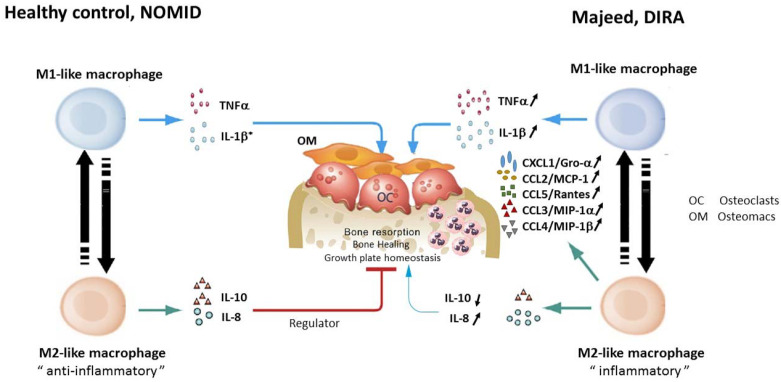
Impaired bone homeostasis as a disease model for the osteomyelitis phenotype in Majeed syndrome. In Majeed syndrome proinflammatory M2-like macrophages produce increased amounts of IL-8 and osteoclastogenic chemokines and lower IL-10 levels thus shifting towards a pro-inflammatory “environment”. The increased production of chemokines such as CXCL1 with IL-8 lead to recruitment of neutrophils while MCP-1 and MIP-1α/β recruit monocytes and affect macrophage differentiation. The chemokines released by the inflammatory M2-like macrophages further propagate osteoclastogenesis leading to the bone destruction seen in Majeed syndrome and DIRA. In contrast, the M1-like macrophages in NOMID (an NLRP3 inflammasomopathy) also produce increased IL-1β, yet the M2-like macrophages are not inflammatory and osteoclastogenesis is not increased thus osteomyelitis is not part of their phenotype. (Reprinted from Bhuyan et al. Arthritis and Rheumatology, 2021—Reference [81]).

Most recently macrophage polarization has been implicated in the sterile osteomyelitis that is a prominent feature of Majeed syndrome. Bhuyan et al. report extensive investigations on cells obtained from the first Majeed syndrome patient who resides in the US who was found to be heterozygous for 2 novel *LPIN2* mutations (Table 1, K1) [81]. As expected, monocytes and monocyte-derived M1-like macrophages from patients with Majeed syndrome as well as monocytes from individuals with another genetically driven NLRP3 inflammasomopathy Neonatal Onset Multisystem Inflammatory Disorder [NOMID] had elevated caspase-1 activity and their cells secreted more IL-1β levels when compared to healthy controls [81]. Yet, only the cells from Majeed syndrome patients (versus NOMID or healthy controls) showed increased expression of osteoclastogenic mediators including IL-8, IL-6, TNF, CCL2, MIP1α, MIP-1β, CXCL8/IL-8 and CXCL1 in M2-like macrophages stimulated with LPS [81]. In addition, Majeed cells showed increased osteoclastogenesis in response to RANKL and M-CSF, associated with higher NFATc1 levels, showed enhanced JNK/MAP kinase activation and reduced Src kinase activation. The data on this single patient suggests that Lipin-2 modulates bone homeostasis through alteration of phosphorylation levels of JNK and Src kinases driving differentiation of macrophages to a pro -inflammatory M2- phenotype and by driving osteoclastogenesis. This could help explain why the bone is triggered in this IL-1 mediated disease but not other IL-1 mediated disorders (Figure 2). Further, these proinflammatory cellular changes could be attenuated by IL-1 inhibitors and JNK inhibitors suggesting that the JNK/MAP kinase pathways may be a novel target for treatment [81].

## 4. Conclusions

In summary, Majeed syndrome is a rare autosomal recessive disorder due to loss of function mutations in *LPIN2*. Low intra cellular cholesterol leads to altered function of the P2X7R and subsequent K+ efflux and NLRP3 inflammasome activation leading to enhance production of proinflammatory cytokines including IL-1. There are no clinical trials in Majeed syndrome, so treatment is empiric. Therapeutically blocking the IL-1RI or IL-1β has been utilized in 10 patients with Majeed syndrome and all have reported significant benefit with resolution of the inflammatory bone disease and normalization of inflammatory markers. Several patients treated with IL-1 blocking agents have had improvement in their anemia but none have had repeat bone marrow biopsies to determine if the dyserythropoeisis which is a classic part of the disease is reversed with IL-1 blockade. Given that chronic inflammation can result in anemia of chronic disease, it remains unclear if the CDA is improved with IL-1 blockade or if simply that a component of the anemia was due to chronic inflammation which resolves with treatment. Further study is needed to determine best treatment for Majeed syndrome. 

## Figures and Tables

**Table 1:** Clinical features in Majeed syndrome.

	A1	A2	A3	A4	B1	B2	C1	D1	D2	E1	E2	F1	F2	G1	H1	H2	I1	I2	I3	I4	I5	I6	J1	K1
**Recurrent fever**	+	+	+	+	+++	+++	+	–	+	–	–	NR	NR	–	+	–	+	NR	NR	–	–	–	+	–
**Failure to thrive**	+	+	+	+	+	+	–	NR	NR	+	–	NR	NR	+	–	–	+	NR	NR	NR	NR	NR	NR	–
**CNO**	+	+	+	+	+	+	+	+	+	+	+	+	+	+	+	+	+	+	+	Joint and bone pain	Joint and bone pain	Knee pains	–	+
Age at onset (months) ^Δ^	12	19	9	1	0.75	9	15	6	3	24	96	6	48	72	13	15	infant	?	?	?	?	?	6	12
LE long bones	+	+	+	+	+	+	+	+	+	+	+	+	+	+	+	+	+							+
UE long bones	+	+	+	+	+	+	+	+	+	+	+	–	–	NR	NR	ND	NR							NR
Feet	–	–	–	–	–	+	+	–	–	+	–	+	–	+	–	ND	NR							NR
Spine ^#^	–	–	–	–	–	–	–	–	–	–	–	–	–	–	ND	ND	NR							NR
Other			hands			hands		ribs	hands											Limb pain	Limb pain	Knee pain		
Bone biopsy	osteo	osteo	osteo	NR	NR	NR	ND	Osteo ^Φ^	ND	ND	ND	ND	necrosis	osteo	c/w osteo	ND	ND	ND	ND	ND	ND	ND	ND	ND
**Objective joint swelling**	+	+	+	NR	+	+	+	–	+	+	+	+	+	+	+	–	NR	NR	NR	NR	NR	NR	–	–
**Hepatosplenomegaly**	+	+	+	+	+	NR	+	–	–	+	–	NR	NR	NR	–	–	NR	NR	NR	NR	NR	NR	–	NR
**↑ ESR/CRP**	+++	+++	+++	+++	++	+++	+++	+++	+++	+++	++	+++	++	+++	++	++	+++	+++	++	NR	NR	NR	+++	+++
**Microcytic anemia**	+	+	+	+	+ 8.8	+ 4.0	+	+°	+°	+	+*	+	+	NR	9.3 MCV	7.7@	6.9	+	+	anemia	anemia	–	8.5	10.1
Transfusion Rx	++	+++	++	++	++	+++	–	–	–	–	–	–	–	NR	–	–	–	–	–	–	–	–	–	–
Dyserythropoiesis on BM	+	+	+	+	+	+	+	+	+	+	ND	ND	+	NR	ND	+	+	ND	ND	ND	ND	ND	–	+
**Neutropenia** **(ANC)**							+ (750)			leuko	–				+ (588)	NR	NR	NR	NR	NR	NR	NR	+ (400)	–
**Neutrophilic dermatosis**	+	+	–	–	–	–	–	–	–	–	–	–	–	NR	–	–	–	–	–	–	–	–	–	–
**Treatments**	Pred, NSAID	Failed colchicine. Pred, NSAID	Failed colchicine. Pred, NSAID	Pred, NSAID	NSAID	NSAID	NSAID + prednisolone	Steroids, Failed etanercept, improved with anakinra, canakin	Steroids, Failed etanercept, improved with anakinra, canakin	NSAId, MTX, partial pam	MTX	NSAID	NSAID, canak	Anakinra	NSAID, Etanercept (failed), did well on anakinra	Failed etanercept, improved with anakinra	NSAID, steroid, partial resonse to bisphos, anakinra	Anakinra	Anakinra	NSAID	NSAID	none	none	NSAID, canakin
**Mutation**	S734L	S734L	S734L	S734L	C181*	C181*	R776Sfs*66	L439fs*15	L439fs*15	Y747*	Y747*	S734L	S734L	R776Sfs*66	R776Sfs*66	R776Sfs*66	R736H	R736H	R736H	R736H	R736H	R736H	R776Sfs*66 And R564Kfs*3	R517H and 17.8 kb del

Osteo = osteomyelitis; ND = not done; NR = not reported; # whole body MRI not done in most; Φ granulomatous inflammation; ° anemic but MCV not reported; * mild anemia MCV 86.8. − = not present; + = present or mildly elevated; ++ = moderately elevated; +++ = markedly elevated. Reference for family A and B (1); family C (4), family D (5), family E (6), two patients reported by Moussa F (7), patient G (8). ^Δ^ months to year conversion: 24 mo = 2 yrs; 48 mo = 4 yrs; 72 mo = 6 yrs; 96 mo = 8 yrs. Modified from Ferguson PJ; Elsevier, Edited by Cimaz and Lehman, Pediatric in Systemic Autoimmune Diseases, Volume 11, Chapter 15, Pages 315-339, 2016. @ dimorphic red cells on peripheral smear (A) Majeed 1989 (B) Majeed 1989 (C) Al-Mosawi 2007 (D) Herlin (E) Rao (F) Moussa (G) Pinto-Fernandez (H) Al-Mosai 2019 (I) Roy 2019 (J) Liu (K) Bhuyan 2020.

**Table 2:** Majeed Clinical Features.

Clinical Feature	%
Recurrent fever	46
Failure to thrive	38
Hepatosplenomegaly	30
Objective limb swelling	54
Neutrophilic dermatosis	8
↑inflammatory marker	88
Microcytic anemia/CDA	92
Neutropenia	13
Radiographic CNO^++^	83

Percentage of cases reporting this feature. Total *n* = 24. ++ No imaging was done in 4 but ¾ had bone pain.
